# Genomic Adaptive Evolution of Sand Rice (*Agriophyllum squarrosum*) and Its Implications for Desert Ecosystem Restoration

**DOI:** 10.3389/fgene.2021.656061

**Published:** 2021-04-30

**Authors:** Chaoju Qian, Xia Yan, Tingzhou Fang, Xiaoyue Yin, Shanshan Zhou, Xingke Fan, Yuxiao Chang, Xiao-Fei Ma

**Affiliations:** ^1^Key Laboratory of Stress Physiology and Ecology in Cold and Arid Regions, Gansu Province, Department of Ecology and Agriculture Research, Northwest Institute of Eco-Environment and Resources, Chinese Academy of Sciences, Lanzhou, China; ^2^School of Life Sciences, Nantong University, Nantong, China; ^3^Key Laboratory of Eco-hydrology of Inland River Basin, Northwest Institute of Eco-Environment and Resources, Chinese Academy of Sciences, Lanzhou, China; ^4^College of Resources and Environment, University of Chinese Academy of Sciences, Beijing, China; ^5^Agricultural Genomics Institute at Shenzhen, Chinese Academy of Agricultural Sciences, Shenzhen, China

**Keywords:** sand rice (*Agriophyllum squarrosum*), environmental heterogeneity, RAD sequencing, balancing selection, desertification reversion

## Abstract

Natural selection is a significant driver of population divergence and speciation of plants. Due to local adaptation to geographic regions with ecological gradients, plant populations harbored a wide range of adaptive genetic variation to enable them to survive the heterogeneous habitats. This is all the more necessary for desert plants, as they must tolerant more striking gradients of abiotic stresses. However, the genomic mechanism by which desert plants adapt to ecological heterogeneity remains unclear, which could help to guide the sustainability of desert ecosystems. Here, using restriction-site-associated DNA sequencing in 38 natural populations, we investigated the genomic divergence and environmental adaptation of sand rice, *Agriophyllum squarrosum*, an annual pioneer species that covers sand dunes in northern China. Population genetic structure analyses showed that sand rice could be divided into three geographically distinct lineages, namely, *Northwest*, *Central*, and *East*. Phylogeographic analyses revealed that the plant might originate locally in Bergen County and further differentiated into the *East* lineage and then the *Central* lineage. Ecological niche modeling found that different lineages occupied distinct ecological niches, suggesting that the ecological gradient would have triggered genomic differentiation among sand rice lineages. Ecological association study supported that the three SNPs under divergent selection were closely correlated with precipitation gradients, indicating that precipitation might be the most important stress trigger for lineage diversity in sand rice. These adaptive SNPs could be used to genotype suitable germplasms for the ecological restoration of specific desertified lands. Further analyses found that genetic structure could significantly overestimate the signals for balancing selection. Within the *Central* lineage, we still found that 175 SNPs could be subject to balancing selection, which could be the means by which sand rice maintains genetic diversity and adapts to multiple stresses across heterogeneous deserts and sandy lands. From a genomic point of view, this study highlighted the local and global adaptation patterns of a desert plant to extreme and heterogeneous habitats. Our data provide molecular guidance for the restoration of desertified lands in the arid and semi-arid regions of China and could facilitate the marker assistant breeding of this potential crop to mitigate climate change.

## Introduction

Natural selection is acknowledged to be the main driver of population divergence and even speciation, contributing highly to the diversity of species (Dobzhansky, 1940; Darwin, 1968; Mayr, 1992; Schneider, 2000). Divergent selection is one of the most important fundamental evolutionary processes for genetic differentiation among populations, and further facilitates reproductive isolation among populations and completes speciation (Thompson, 2016; Bamba et al., 2019; Li J. L. et al., 2020). Numerous studies of plants have found that understanding local population adaptations could not only provide more information on how populations respond to divergent ecological factors but could also provide far-reaching implications for species management, conservation, and ability to cope with global climate change (Fady et al., 2016; Lázaro-Nogal et al., 2016; Rellstab et al., 2016; Escalante et al., 2020; Li L.-F. et al., 2020; Li Y. et al., 2020; Moran, 2020). The consequences and mechanisms of balancing selection in plants have seldom been addressed due to the partial or lacking genomic investigation of plant populations (Zou et al., 2017), with the exception of information on biotic interactions such as sex-determining alleles among model plants (Weis et al., 2017). However, the roles of diversifying selection and balancing selection have not been fully discussed in pioneer species for the conservation and restoration of fragile ecosystems, especially for desert ecosystems that are facing multiple stresses and climate change (Swart et al., 1998; Leemans and Eickhout, 2004).

Characterized by low biodiversity and high environmental stress, desert ecosystems are believed to be much more sensitive to climate change than other ecosystems. Thus, under conditions of rapid climate change, the sustainability of desert ecosystems is more at risk (Thomas et al., 2005; Wang et al., 2009). Furthermore, due to the long-term stresses of excessive grazing and intensive cultivation, fragile desert ecosystems have been subjected to severe erosion and land degradation (Lu et al., 2018). To prevent land degradation and eco-environmental deterioration, the Chinese government has, since 1979, successively implemented a series of ecological construction projects aimed at the restoration of desertified land, such as the Three North Shelterbelt Project, Converting Farmland to Forests, Beijing-Tianjin Sand Source Control, Grain-for-Green Program, and Key Management of Shiyang River Basin (Feng et al., 2016; Lu et al., 2018; Yin, 2018). Unfortunately, because of their poor adaptability to the local environment, these large-scale artificial forests began to decline a few decades later since afforestation (Jiang et al., 2006; Cao et al., 2010). Thus, native plant species/populations with local adaptations should be chosen to enable successful desert restoration.

Deserts in marginal monsoonal zones tend to experience frequent contractions and expansions during glacial and inter-glacial periods (Lu et al., 2013), during which times the dynamics (such as the distribution ranges and effective population size) of desert plants in this region are significantly sped up (Yin et al., 2015; Qian et al., 2016), which sheds an interesting light on the influence of climate change on the demographic history of desert plant species (Wang et al., 2013; Ignace et al., 2018; Shi et al., 2020; Yin et al., 2020). During long-term adaptation to monsoonal climate change and heterogeneous environments, those populations can be expected to harbor more valuable genetic resources. To exemplify the ways that their potential genetic resources can be evaluated across natural plant populations in different geographical and climatic zones, it is critical to fully investigate the adaptive genomic patterns within pioneer species widely distributed across desert areas. The knowledge of the molecular mechanisms for desert plants adapting to heterogeneous environments can guarantee the successful restoration of the desert ecosystems.

Sand rice (*Agriophyllum squarrosum*, Chenopodiaceae) is an annual plant species that is widely distributed across the sand dunes of all of the deserts and sandy lands across the Asian interior^1^. It can survive extremely high temperatures, drought, and sand burial (Li et al., 1992; Miao et al., 2013; Zhao et al., 2014). As a primary and pioneer species in the reversal of desertification, sand rice plays a key role in the succession of fragile desert ecosystems, which is a rich source of carbon and nitrogen in such poor soil environments and the withered plants can also reduce at least 90% of wind velocity (Ma et al., 2008; Chen et al., 2009). Sand rice also has high edible and medicinal values. Its grains provide rich and balanced nutrition that is comparable to *Chenopodium quinoa*, an important food resource recommended by the United Nations Food and Agriculture Organization (Chen et al., 2014). The above-ground tissues of sand rice are also rich in the active ingredients of alkaloids, polyphenols, and flavonoids, which have anti-oxidation and anti-inflammatory effects (Yin et al., 2018). Thus, this plant is believed to be an invaluable candidate species for domestication as an ideally nutritious food and non-resistant forage crop that can help mitigate future climate change. Study of multiple cpDNA fragments and nuclear ITS has shown that sand rice has significant differences in genetic structures among its geographical populations (Qian et al., 2016). Common garden experiments on sand rice have also shown that their phenotypic traits are significantly differentiated among the natural populations in a way that is highly correlated with local environmental and climatic factors (Yin et al., 2016a, b). Because only a few neutral molecular markers were used in previous studies, the fine genetic structure and mechanisms of the adaptive genomic differentiation of sand rice is still far from clear.

In this study, using restriction-site associated DNA (RAD) sequencing, we investigated population genomic diversity in natural populations of sand rice collected from almost all of the deserts and sandy lands of northern China. By precisely describing the geographical variation in genetic structure, thoroughly profiling the shifts in distribution region for each genetic lineage coordinated with changes in climate in the past and future and combining associations between genetic variation and the gradients of the ecological factors of the *in situ* populations, we elucidated the following issues that might affect the successful restoration of desert ecosystems: (i) because the colonization of sand rice was accompanied with the shifting of sand dunes, there existed diverse hydrothermal heterogeneity among the different deserts, so it was investigated whether the genomic differentiation in sand rice was triggered by ecological heterogeneity; (ii) it was also examined how diversifying and/or balancing selection shaped genomic differentiation among the genetic lineages, as well as whether the allele frequency of these adaptive SNPs was associated with the ecological gradient. The clarification of these questions could shed light on the construction of ideal founder populations in specific regions during desert restoration. This could also provide germplasms for breeding high-resistance plants to cope with future global climate change.

## Materials and Methods

### Sample Collection and DNA Extraction

**FIGURE 1:**
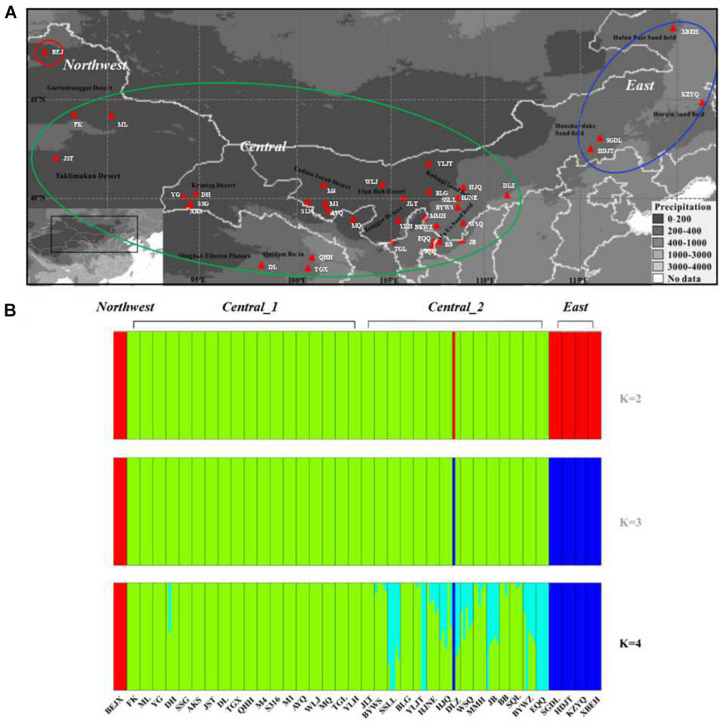
Geographic distribution and genetic clusters of the 38 sand rice populations used in this study. **(A)** Geographic distribution of the sampled sand rice populations. Populations in the red circle represent the populations were grouped into the *Northwest* lineage, populations in the green circle represent the populations were grouped into the *Central* lineage, populaitons in the blue circle represent the populations were grouped into the *East* lineage. **(B)** Population structure of the 38 sand rice populations inferred using FastStructure, based on neutral SNPs.

A total of 187 individuals from 38 populations (3--5 individuals for each population) were collected across all deserts and sandy lands in the Asian interior, as indicated by the distribution range of sand rice from the Chinese Virtual Herbarium^2^ and field records (Qian et al., 2016; Figure 1 and Supplementary Table 1). Fresh leaves were dried and preserved in silica gel, and voucher specimens were deposited at the Key Laboratory of Stress Physiology and Ecology in Cold and Arid Regions, Gansu Province, Northwest Institute of Eco-Environment and Resources, Chinese Academy of Sciences. The total genomic DNA was isolated from the tissue samples with a MagCore Genomic DNA Plant Kit via MagCore^®^ automated extraction instrument following the manufacturer’s protocol. All of the DNA quantity was assessed with Qubit assay HS kit (Life Technologies, Burlington, ON, Canada) using a Qubit v2.0 (Life Technologies).

### Library Construction and RAD Sequencing

After being examined by both double digest and single digest with different restriction enzymes, *Eco*RI single digest RAD libraries were prepared, following the protocol of Baird et al. (2008) for each individual. In brief, 1 μg genomic DNA was digested for 3–4 h at 37°C in a 40 μL reaction with 20 units of *Eco*RI restriction enzyme (specific for the G| AATTC motif) per sample. Then the digested and purified DNA was ligated to P1 barcoded adapters and was sheared to an average size of 350 bp using a Bioruptor pico. After P2 adapter ligation, to remove the adapter dimers, libraries with DNA 300–500 bp were further purified using the Qiagen Mini Elute Kit (Qiagen) according to the protocol of the manufacturer. All of the libraries were enriched with PCR amplification using KAPA Library Amplification Kits, purified again with the Qiagen Mini Elute Kit, and sequenced on a HiSeq 3000 platform (Illumina, San Diego, CA, United States) as 150 bp paired end sequences with one library per lane. The sequences generated by Illumina HiSeq 3000 are available in the NCBI Sequence Read Archive (SRA) under the accession number PRJNA693348.

### Raw Data Filtering and SNP Calling

Raw reads were demultiplexed using *process_radtags* in Stacks Version 1.48^3^ and the quality for each sample was checked using FastQC^4^. The raw reads were filtered for quality, length, and ambiguous barcodes by Skewer (Jiang et al., 2014). All of the remaining reads from each individual were further used for genotyping and SNP calling with *de novo* pipeline in STACKS v.0.9999 (Catchen et al., 2011). Putative orthologous loci were assembled using ustacks with a preliminary dataset set using a minimum depth of coverage to create a stack (*-m*) of 15. The maximum number of mismatches between two stacks in a locus in each sample (*-M*) was seven. The catalog of loci was assembled using *cstacks* with a distance allowed between loci in the catalog (*-n*) of five. Then, PLINK v.1.07 was used to filter the final dataset with the following criteria: (i) putative polymorphic SNPs in one population were present in all of the samples of this population and should be presented in at least 5 of the total 38 populations; (ii) to minimize the physical linkage, only one SNP was remained for per RAD tag; and (iii) SNPs with more than two alleles were discarded.

### Population Genetic Diversity, Divergent Loci, Genetic Structure, and Phylogenetic Relationship Analyses

To investigate the general genetic diversity of sand rice, we used the resulting full SNP dataset with SNPs from all frequencies to estimate genetic diversity statistics, such as number of segregating sites (S), average pairwise differences (π) and Waterson’s θ (θw) in each population and geographic group, using DnaSP version 5.10.01 (Librado and Rozas, 2009).

Previous studies that used neutrality markers (ITS and five cpDNA fragments) showed significant genetic differentiation among the geographical populations of sand rice from northern China (Qian et al., 2016). Thus, to dissect the genetic basis for local adaptation among sand rice populations, two methods were used to detect the selection signals resulted by environmental heterogeneity. First, we adopted the widely used *F*_ST_-based outlier approach based on the multinomial Dirichlet model, implemented in BayeScan, version 2.1 (Foll and Gaggiotti, 2008). Compared to those neutral SNPs, SNPs under diversifying selection should have higher values for *F*_ST_, while balancing selection should result in lower *F*_ST_ values (Beaumont and Nichols, 1996; Foll and Gaggiotti, 2008). Second, we also used logistic regression models to identify the possible locus associated with environment variables with Samβada (Stucki et al., 2017). The multivariate option was chosen for data analyses, as this allows a combination of predictor variables to be simultaneously assessed and could reduce the occurrence of spurious genotypes through environment associations (Stucki et al., 2017). A total of 19 independent environmental variables at a resolution of 2.5 arc-minutes were downloaded from the WorldClim database version 1.4 (1950--2000)^5^, and altitude, longitude, and latitude were taken for each population for the association analyses. The significance of the outputs was assessed taking the log-likelihood ratios (*G*-scores) into account and was provided as the *P*-values derived from the χ^2^ test (Joost et al., 2007).

Then, based on multiple neutral SNPs but excluding SNPs that were involved in local adaptation, as described above, a Bayesian algorithm implemented in FASTSTRUCTURE (Raj et al., 2014) was used to estimate the genetic clustering (*K* = 2–8) with 10 replicates of 1,000,000 iterations for each *K*-value. The results were further analyzed with the choose*K*.py script, and *K* = 4 was determined to be the model that best explained the variation in the data. PCA (Maćkiewicz and Ratajczak, 1993) and the FineRADStructure (Malinsky et al., 2018) were also used to check the genetic clusters. Furthermore, INSTRUCT (Gao et al., 2007) was also run for *K* = 2–8 in mode 2 for the joint inference of the population-selfing rate and the population substructure, as sand rice might have a high selfing rate (Qian et al., 2016).

The historic event model of the sand rice was reconstructed with ^∗^_BEAST_ version 2.4.1 (Bouckaert et al., 2014) to reflect the divergence history of each sand rice lineage. The BEAUti function implemented in ^∗^_BEAST_ was used to generate an input file with 5,831 neutral SNPs. A strict clock was used as the molecular clock, and the Yule model was used as the speciation prior. The best substitution model for these combined 5,831 SNPs was estimated using jModelTest version 2.1.7 (Posada, 2008). Three independent runs were carried out with the Markov chain Monte Carlo. The length was set to 80 million generations, and trees were stored every 8000 generations. Then, Tracer version 1.7.1 (Drummond and Rambaut, 2007) was used to ensure that each run converged to a similar stationary distribution, and LogCombiner version 1.7.4 (Drummond and Rambaut, 2007) was used to combine the log and tree files for each run with 10% burn-in. Finally, the maximum lineage credibility tree was summarized from consensus trees with TreeAnnotator version 1.7.5 (Drummond and Rambaut, 2007).

### Ecological Niche Modeling and Identity Testing

Based on our field-work records and documents from the Chinese Virtual Herbarium (see text footnote 2), 103 non-duplicate data points were used to estimate the distribution regions, composed of 89 and 14 points for the *Central* and *East* lineages, respectively. Due to the small distribution for the *Northwest* ancestral lineage, with only one point, to avoid bias, this lineage was excluded from the ecological niche modeling (ENM). After removing those highly correlated bio-climatic variables (i.e., those with Pearson correlation coefficients >0.9) among the 19 climatic variables from the WorldClim database (Hijmans et al., 2005) using ENMTools version 1.4.3 (Warren et al., 2010), twelve environmental variables were retained for the subsequent analyses. The present distribution region for each lineage was estimated by maximum entropy modeling with MAXENT version 3.4.1 under 100 replicates of cross-validation with default settings (Phillips et al., 2006). The model accuracy was evaluated using the area under the receiver operating characteristics curve (AUC), which gives values from 0 (no discrimination) to 1 (perfect discrimination), where a score of 0.5 suggests that the discriminatory power of the model is no better than random prediction (Elith et al., 2006). Then the identity tests were applied to compare the similarity of the distribution models of the two lineages. First, the null hypothesis proposed that each lineage pair is distributed in an identical environmental area. Then, Schoener’s D similarity index (Schoener, 1970) and Warren’s I measure of niche overlap (Warren et al., 2008) were calculated with ENMTools. Finally, to test the significance of the divergence between the distribution models, 100 simulations were carried out for each lineage pairwise comparison. The current model was also projected into the past (LIG, ∼140 Kya and LGM, ∼21 Kya) and future (the year 2070 with moderate carbon release was chosen based on the average between the 2061 and 2080) layers to predict the potential distribution range shifts of sand rice in response to climate change.

### Isolation by Distance and Isolation by Environment Testing

To evaluate the effect of geographical distance among these populations on the genetic divergence, IBD (isolation by distance) were assessed across all populations by comparing the matrices of geographic and genetic distances. In addition, to verify the effect of ecological distance on the genetic structure, IBE were also estimated among all these populations by comparing the matrices of ecological (converted to a binary factor) and genetic distance. Nineteen environmental variables at a resolution of 2.5 arc-minutes from the WorldClim database version 1.4 (1950–2000, see text footnote 5) were used to calculate the ecological distance. The genetic distances among populations were calculated with Arlequin version 3.11 (Excoffier et al., 2005). Then, to assess the statistical correlation among matrices, we applied Mantel tests with 9999 randomizations between geographic and genetic distance and between ecological and genetic distance using the *dist_amova* function in the GSTUDIO R package (Dyer and Nason, 2004) and *mantel.partial* function in VEGAN R package (Oksanen et al., 2015) among populations.

## Results

### SNP Discovery and Overall Genetic Variation

Restriction-site associated DNA sequencing was used to investigate the genome-wide diversity of sand rice, and the final sample set included 187 individuals who represent 38 populations (Figure 1 and Supplementary Table 1). After removing the low-quality reads and samples, we obtain a total of 666.68 million reads for 187 individuals. For each individual, the number of raw sequences reads ranged from 1.06 to 9.23 million, and the mean coverage depth ranged from 17× to 45×. On average, 94.96% of the reads were utilized in the *de novo* assembly of the RAD catalog, and 306,276 high quality catalogs with polymorphic sites and 1,102,051 SNPs were assembled and identified, respectively. After *de novo* catalog building and SNP calling filtered with those strict criteria, 6,124 SNPs were finally identified and used for further analyses.

The genetic diversity analyses shown in Table 1 showed that the number of segregate sites ranged from 2 (the HDJT and KZYQ populations) to 543 (the DLZ population) per population. Waterson’s θ (θ_W_) was estimated to be 0.0120 (the KZYQ population) to 0.1340 (the DLZ population) for each population, and nucleotide diversity (π) ranged from 0.0103 (the SGDL population) to 0.1582 (the DLZ population). The number of haplotypes for each population ranged from 3 to 10, and the haplotype diversity ranged from 0.622 to 1.000. In general, the genetic diversity of the four populations from the sandy lands of Hunshandake, Horqin, and Hulun Buir were lower than those of populations. Further, pairwise genetic differentiation was significantly higher between these four populations and other populations (Supplementary Table 2).

**TABLE 1:** Grouping and summary of genetic diversity of each population in *A. squarrosum*.

**group**	**No.**	**Population code**	***N***	***s***	***h***	**Hd**	**π**	**θ_W_**
BEJ	1	BEJX	5	30	9	0.978	0.0425	0.0307
	2	FK	5	54	9	0.978	0.0249	0.0205
	3	ML	5	50	9	0.978	0.0397	0.0382
	4	YG	5	178	10	1.000	0.0188	0.0177
	5	DH	5	320	10	1.000	0.0392	0.0382
	6	SSG	5	70	10	1.000	0.0514	0.0462
	7	AKS	5	334	10	1.000	0.0417	0.0377
	8	JST	5	7	7	0.911	0.0152	0.0151
	9	DL	5	157	10	1.000	0.0308	0.0268
	10	TGX	5	15	10	1.000	0.0208	0.0176
	11	QHH	5	19	8	0.933	0.0197	0.0188
	12	M4	5	9	8	0.933	0.0160	0.0191
	13	S136	5	83	10	1.000	0.0376	0.0384
	14	M1	5	8	9	0.978	0.0311	0.0321
	15	AYQ	5	37	9	0.978	0.0303	0.0315
	16	WLJ	5	17	10	1.000	0.0321	0.0328
	17	MQ	5	15	7	0.867	0.0189	0.0204
Central	18	TGL	5	29	10	1.000	0.0256	0.0240
	19	YLH	5	19	10	1.000	0.0207	0.0198
	20	JLT	5	16	8	0.933	0.0219	0.0255
	21	BYWS	5	115	10	1.000	0.0529	0.0515
	22	SSLL	5	259	10	1.000	0.0497	0.0449
	23	BLG	5	168	10	1.000	0.0624	0.0590
	24	YLJT	5	462	10	1.000	0.0652	0.0586
	25	HJNE	5	169	10	1.000	0.0584	0.0525
	26	HJQ	5	90	10	1.000	0.0538	0.0549
	27	DLZ	3	543	6	1.000	0.1582	0.1340
	28	WSQ	5	34	10	1.000	0.0496	0.0473
	29	MMH	5	8	9	0.978	0.0165	0.0179
	30	JB	5	25	10	1.000	0.0349	0.0306
	31	BB	4	43	8	1.000	0.0409	0.0370
	32	SQL	5	16	9	0.978	0.0238	0.0264
	33	BYWZ	5	15	8	0.933	0.0350	0.0336
	34	EQQ	5	81	10	1.000	0.0210	0.0203
		Average	–	–	–	–	0.0381	0.0360
East	35	SGDL	5	101	10	1.000	0.0103	0.0088
	36	HDJT	5	2	3	0.622	0.0192	0.0191
	37	KZYQ	5	2	3	0.711	0.0151	0.0120
	38	XBEH	5	113	10	1.000	0.0191	0.0153
		Average	–	–	–	–	0.0159	0.0138

**N*: Number of individuals; *s*: Number of segregating sites; *H*: Number of Haplotypes; π: Nucleotide diversity; Hd: Haplotype diversity; θ_*W*_:Waterson’s θ.*

### SNPs Outliers With Genetic Structure

**FIGURE 2:**
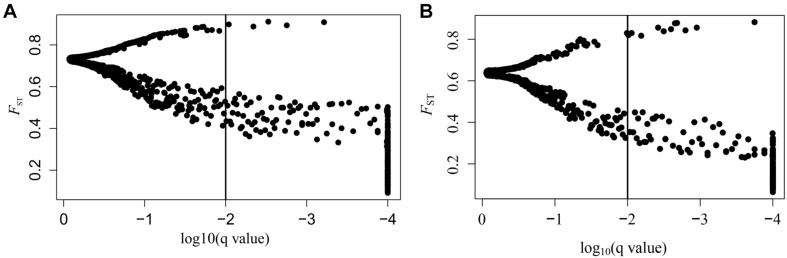
Results of BayeScan. **(A)** *F*_ST_ outliers of all *A. squarrosum* populations sampled in this study. The *x*-axis represents the *q*-value, which is standardized to log10, and the *y*-axis represents *F*_ST_ values. **(B)** *F*_ST_ outliers of sand rice populations of the *Central* lineage. The *x*-axis represents the *q*-value, which is standardized to log10, and the *y*-axis represents *F*_ST_ values.

**FIGURE 3:**
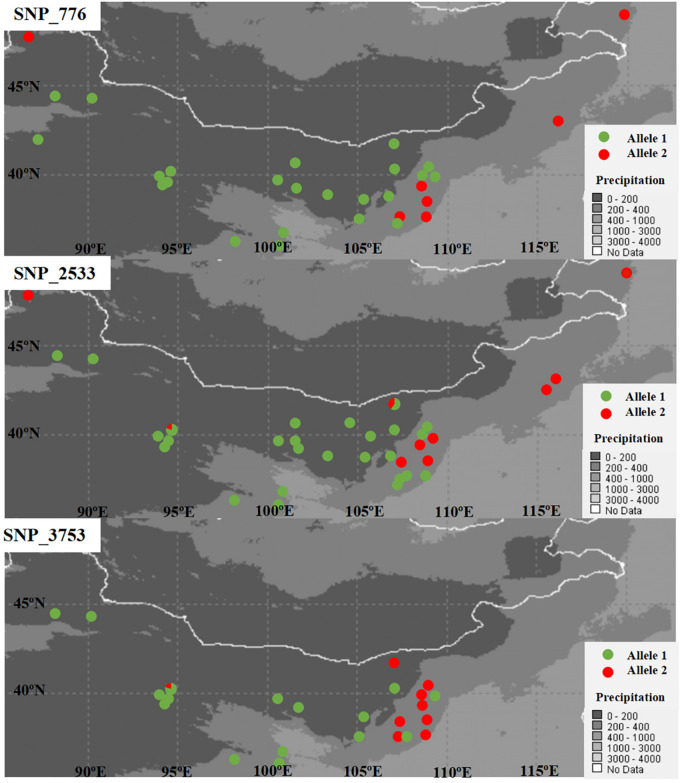
Plots of allele frequency in each population for the three detected SNPs under diversifying selection.

SNPs with extreme allele frequency differences across populations were identified using *F*_ST_ outlier analyses to further explore the potential candidate loci under selection, and 243 outlier SNPs across all populations were detected, with a false discovery rate of 1% (Figure 2A). Among the 243 outlier SNPs, according to the criteria of BayeScan, 5 were suggested to be subject to diversifying selection, and the other 238 were subject to balancing selection. On the other hand, 717 associations between 293 SNPs outliers and 22 climate/geographic variables were identified with Samβada software by *P* < 0.0001 (Supplementary Table 3). Among these, 328 associations were related to temperature, 275 to precipitation, 69 to latitude, 35 to longitude, and 10 to altitude (Supplementary Table 3). The five divergent selected outlier SNPs (SNP_776, SNP_2533, SNP_3753, SNP_5540, and SNP_6016) detected by BayeScan were further identified to be associated with climate/geographic variables, and four were significantly associated with precipitation, with the exception of SNP_5540, which was associated with annual mean temperature (Supplementary Table 3). Interestingly, as shown in Figure 3, allele frequency distribution analyses of these five outlier SNPs showed a significant differentiation between semi-arid populations and those from arid regions of northern China.

To investigate the genetic structure of the genomic variation, 5831 neutral SNPs were used to construct the genetic structure of the sand rice populations. The results of FASTSTRUCTURE predicated *K* = 4 as the optimum population structure for *A. squarrosum* populations. As shown in Figure 1B, the four groups were divided as follows: (1) *Northwest*, including only one population of BEJ, the ancestral population of sand rice as suggested by the results of FineRADStructure and the ^∗^_*B*__*EAST*_; (2) *Central*_1, including 18 populations from the Gurbantünggüt desert, the Taklamakan desert, the Kumtag desert, the Qaidam, Badan Jaran desert, and the Tengger desert; (3) *Central*_2, including 15 populations from the Ulan Buh sandy land, the Kubuqi desert, and the Mu Us sandy land, which are located in monsoonal zones; and (4) *East*, including four populations from the Hunshandake sandy land, the Horqin sandy land, and the Hulun Buir sandy land. Two components were found in the group UKM, of which one is unique to this group, while the other one is similar to that in the *Central* lineage. We also found an odd phenomenon in population DLZ; among the three individuals in this population, one individual shared the its genetic component with the *East* lineage, which also contributed to the high values of genetic diversity of this population (Table 1). To avoid possible sampling errors or sample contamination, we deleted this population from the analyses. On the other hand, pairwise genetic distance analyses showed that lineages of *Central*_1 and *Central*_2 lineages were closely related due to the frequent gene flow (*F*_ST_ = 0.01, *P* < 0.01, Supplementary Table 4). The results of PCA, FineRADStructure and INSTRUCT also supported that the *Central*_1 and *Central*_2 lineages were closely related and that there were three genetic lineages among all of the populations (Supplementary Figure 1). Thus, in the further analyses, two groups were merged into one lineage called *Central*. Phylogenetic analyses further supported the conclusion that sand rice may have originated in the far northwest of the Gurbantünggüt Desert (in Burgin County) and then further differentiated into *East* and *Central* lineages (Supplementary Figure 1).

On the other hand, as shown in Figure 2B, when *East* and *Northwest* lineages excluded, significant selection signals were identified in 186 SNPs among populations from the *Central* lineage, with 11 SNPs were under diversifying selection, while the 175 SNPs were remained under balancing selection. Among these 11 SNPs with diversifying selection, three SNPs (SNP_776, SNP_2533, and SNP_3753) were also detected in the upper analyses based on all populations with distinctly genetic structure, while all of the 175 SNPs with balancing selection could also be found in the upper analyses.

### Ecological Niche Modeling and Identity Tests

**FIGURE 4:**
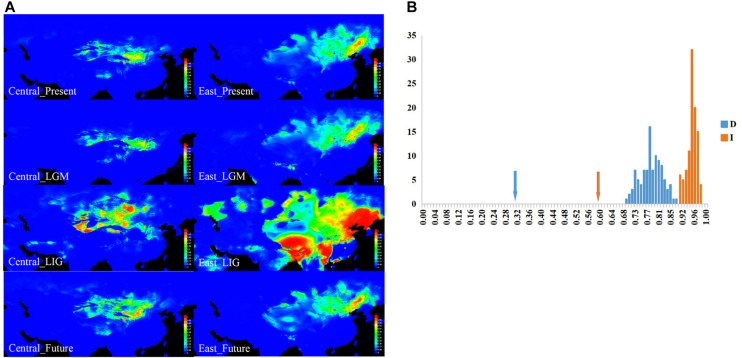
The results for ecological niche modeling suggest three significant divergent niches of sand rice. **(A)** Geographic projections of the *Central* and *East* lineages of sand rice niche derived from the MaxEnt model. These figures originated from the software packages of MAXENT version 3.3.3, the environmental variables originated from the WorldClim database version 1.4 (1950–2000, http://www.worldclim.org/) and then modified by CQ with CorelDraw X6 (Corel Corporation, Ottawa, ON, Canada). **(B)** Results of identity tests. Observed niche overlap values of Schoener’s *D* and *I* for *Central* and *East* lineages compared to null distributions (bars indicate the null distributions of *D* or *I*). All pairwise comparisons show significant niche divergence (*P* < 0.05).

All of the AUC scores are over 0.9 (0.977 and 0.977 for *Central* and *East* lineages’ ecological niche prediction, respectively), supporting the suitability of our models. The results of ENM indicated that the *Central* and *East* lineages occupied distinct niches (Figure 4A). For the *Central* lineage, precipitation, the mean temperature of the coldest quarter, and the mean temperature of the driest quarter were found to have large effects on its distribution ranges. For the *East* lineage, however, besides the above two ecological factors, precipitation seasonality and isothermality were also thought to affect its distribution range (Supplementary Table 5).

Furthermore, ecological identity tests of lineages pairwise based on the Schoener’s *D* and *I* values (Figure 4B) showed that both of these lineages significantly differed from the random distribution (*P* < 0.01), suggesting that the niches occupied by the different lineages were not identical, and the two lineages were somewhat eco-geographically isolated. Furthermore, as illustrated in the ecological niche simulations for the present and LGM periods, the potential distribution regions of the two lineages were separated with small overlap, which suggested that the distribution range of the lineages was not be enlarged during global cooling. Meanwhile, both distribution ranges may have greatly expanded during global warming (LIG and future), particularly for the *East* lineage during the LIG period (Figure 4A and Supplementary Table 5).

Furthermore, besides the influence of the physical distances on the genetic distance for sand rice populations (Mantel test geographical distance vs. genetic distance: *r* = 0.4622, *p*-value = 0.001), divergent ecological factors were also suggested to have significant affection on the genetic structure (Mantel test ecology distance vs. genetic distance: *r* = 0.3487, *p*-value = 0.003) (Supplementary Figure 2), suggested that the ecological divergence would also triggered the genomic divergence of these genetic lineages of sand rice.

## Discussion

China is one of the largest countries affected by desertification. In northern China, more than two million square kilometers is characterized as desert and/or sandy lands (Wang et al., 2008). An annual pioneer and primary desert species, sand rice is an ideal plant for sand fixation and the reversion of desertification. To investigate how to construct ideal founder populations for desert restoration, we investigated the genomic diversity of sand rice based on RAD sequencing. By elucidating the effects of genetic structure on adaptive signal detection, we examined the molecular basis for sand rice adaptability to heterogeneous sand dune environments across the arid and semi-arid regions of north China. Then we discussed the guidelines for artificial vegetation using sand rice for the sustainability of desert ecosystems.

Using RAD sequencing in 38 populations with species-wide sampling, we found a total of 6,124 SNPs without strong LD. To our surprise, we detected only five SNPs involved in local adaptation. Then, based on these SNPs, three distinct genetic lineages were clustered in this study (Figure 1 and Supplementary Figure 1), and sand rice might originate locally in Burgin County and further differentiated into the *East* lineage and then the *Central* lineage (Supplementary Figure 1), which is concordant in the previous study based on nrITS and several cpDNA fragments (Qian et al., 2016). Because high genetic structure affects the signal detection of diversifying selection, particularly in a highly selfing plant (Qian et al., 2020), the genetic structure could be expected to increase the average *F*_ST_ of the genomic background and make it more difficult to detect significant outliers under conditions of spatially diversifying selection. However, the high average *F*_ST_ of the genomic background would have little influence on the detection of SNPs under balancing selection, with have lower *F*_ST_ values than the average *F*_ST_ of the genomic background (Beaumont and Nichols, 1996; Foll and Gaggiotti, 2008). Thus, we analyzed adaptive SNPs by excluding the influence of genetic structure by excluding the *Northwest* and *East* lineages. Ultimately, we detected 11 SNPs under significant diversifying selection in the *Central* populations. Three SNPs (SNP_776, SNP_2533, and SNP_3753) were outliers in both tests (with or without the *East* and *Northwest* lineages). Furthermore, the distribution of their allele frequencies were also significantly associated with precipitation gradients (Supplementary Table 4), suggesting that these SNPs could be used as the characteristic markers for specific population construction in artificial desert restoration. In other research fields, such as forestry management and breeding, adaptive SNPs are commonly used as molecular markers for afforestation (Ma et al., 2010; Hall et al., 2011; Chen et al., 2012, 2016). However, no similar work had been begun in the desert restoration until this study.

To our surprise, 175 out of 6,124 SNPs (∼2.9%) were detected under balancing selection in both the overall samples and the *Central* lineage populations alone. Normally, balancing selection is able to maintain genetic diversity in a population via two key mechanisms: heterozygote advantage and frequency-dependent selection, which contributes to the adaptive potential of a population in the presence of environmental heterogeneity (Barrett and Schluter, 2008). Considering to the high selfing rate of sand rice (∼0.67, estimated by Instruct based on RAD sequences, data not shown), we proposed that a high percentage of balancing selection in the sand rice genome could be due more to frequency-dependent selection than to heterozygous advantage.

In fact, genetic structural data showed that significant amounts of new genetic components exist across populations from the monsoonal zone of the *Central* lineage (Figure 1B). Due to the seesaw battles between summer and winter monsoons during the Pleistocene climatic oscillations (Hallatschek et al., 2007; Excoffier and Ray, 2008), populations from the monsoonal zone could have experienced frequent population bottlenecks and habitat fragmentation, as found in other desert plants (Yin et al., 2015; Shi et al., 2020), which promoted the fixation of new alleles within these local populations and further contributed to complicated genetic components of populations from the monsoonal zone. It is a pity, however, as the RAD reads were too short to be annotated without the genomic sequences of sand rice, that we did not find any genes with their function annotated. We proposed that these SNPs under balancing selection may be linked to the genes functioning in respond to multiple stresses or climatic oscillations.

Numerous studies have found that in glacial periods, such as the LGM, northern China experienced significant desertification (Ding et al., 2005; Lu et al., 2013). As a pioneer desert plant, the colonization of sand rice is accompanied with an expansion of desertification. However, ENM results showed that under global cooling, such as at the present and during the LGM period, the suitable habitats for the *Central* lineage and *East* lineages were quite stable. This suggests that although desertification with global cooling could produce more sand dunes, the habitat of sand rice was not enlarged because of the limitation of its ecological niche. In cases of global warming, such as during the LIG and under a scenario of moderate CO_2_ emissions, the suitable habitats of these two lineages become greatly enlarged, particularly for the *East* lineage. However, for *Central* lineage (average π = 0.0381, Table 1), the genetic diversity of the *East* lineage is fairly low (π = 0.0159, Table 1), and its genetic background did not support a large expansion of its distribution range. Thus, the prediction of ecological niches could be overestimated based solely on 19 climate factors, which would further result in incorrect prediction of the potential distribution ranges. According to this point of view, we should be very careful to narrowly identify the strategy for desertification reversion based on the ENM without genetic evaluation. Besides, other ecological factors such as soil type and wind system should also be included in the description of ecological niches of plant species, at least in desert plants.

Thus, based on a thorough investigation of the genomic diversity and ecological factors of sand rice, an annual pioneer plant species widely endemic to sand dunes in arid and semi-arid regions of China, to build up ideal founder populations for the restoration of the desert ecosystem, we suggest the following. First, we should select native and widely distributed plant species with diverse levels of ecological adaptability. Second, by investigating their genetic structure and screening the SNPs involved into ecological adaptation, we can easily classify genetic lineages by core germplasms or ecotypes. Third, by combining genetic data with ENM to evaluate the risk for each lineages facing the climate change and environmental heterogeneity, the composition of the founder populations can be determined for specific environmental conditions. Finally, common garden and/or transplant experiments can be conducted to verify the fitness of founder populations, which could be exploited to obtain the successful restoration of a desert ecosystem. We have not reached this step yet. Of course, to obtain a more comprehensive understanding of how sand rice can be adapted to the heterogeneous local environment and to global climate change, *de novo* genome sequencing of populations is still needed for future study, which will provide more genetic information and guidance for the resistance and restoration of fragile desert ecosystems as global warming proceeds in the future.

## Data Availability Statement

The datasets presented in this study can be found in online repositories. The names of the repository/repositories and accession number(s) can be found in the article/Supplementary Material.

## Author Contributions

X-FM conceived and designed the investigations. CQ and XYa analyzed the data and wrote the first version of the manuscript. CQ collected the data. TF, XYi, SZ, and XF performed the experiments. X-FM and YC provided improvements to the manuscript. All authors contributed to the article and approved the submitted version.

## Conflict of Interest

The authors declare that the research was conducted in the absence of any commercial or financial relationships that could be construed as a potential conflict of interest. The reviewer YL declared a past co-authorship with one of the authors X-FM, to the handling editor.

## Footnotes

1 http://www.iplant.cn

2 https://www.cvh.ac.cn

3 http://catchenlab.life.illinois.edu/stacks/

4 http://www.bioinformatics.babraham.ac.uk/projects/fastqc

5 http://www.worldclim.org/
