# Supplementary material for: Genomic Adaptive Evolution of Sand Rice (Agriophyllum squarrosum) and Its Implications for Desert Ecosystem Restoration
Source: Front Genet. 2021 Apr 30;12:656061. doi: 10.3389/fgene.2021.656061 (PMC8120313; doi:10.3389/fgene.2021.656061)
Supplement: Supplementary file 1 [file Presentation_1.zip › Supplementary Figure 2.docx]

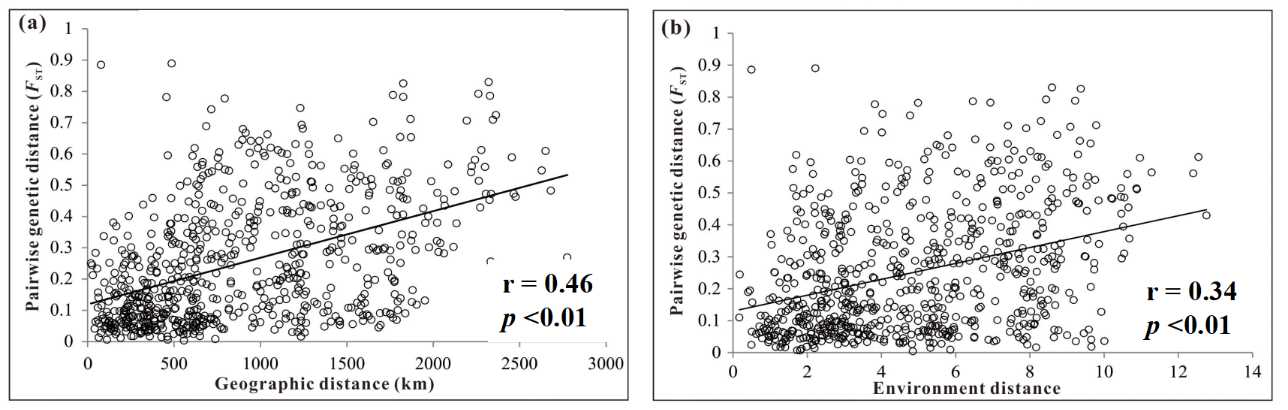


Fig. S2 Results of isolation by distance (IBD) and isolation by environment (IBE) testing. (a) Mantel test of geographical distance *vs* genetic distance among all populations, (b) Mantel test of environmental distance *vs* genetic distance among all populations.
