# Supplementary material for: Genomic Adaptive Evolution of Sand Rice (Agriophyllum squarrosum) and Its Implications for Desert Ecosystem Restoration
Source: Front Genet. 2021 Apr 30;12:656061. doi: 10.3389/fgene.2021.656061 (PMC8120313; doi:10.3389/fgene.2021.656061)
Supplement: Supplementary file 1 [file Presentation_1.zip › Tables & Figures.docx]

**Table S1 Details of the locality information of the sampled populations.**

| **No.** | **group** | **Population code** | **Location**  **(All in China)** | **Latitude**  **(N°)** | **Longitude**  **(E°)** | **Altitude**  **(m)** |
| --- | --- | --- | --- | --- | --- | --- |
| **1** | BEJ | BEJX | Buerjinxi, Gurbantunggut | 47°39′26.48″ | 86°36′20.82″ | 971 |
| **2** | Central | FK | Fukang, Gurbantunggut | 44°22′17.48″ | 88°8′31.20″ | 460 |
| **3** |  | ML | Mulei, Gurbantunggut | 44°14′25.93″ | 90°8′33.84″ | 741 |
| **4** |  | YG | Yangguan, Kumtag | 39°57′37.80″ | 93°59′56.34″ | 1223 |
| **5** |  | DH | Dunhuang,Kumtag | 40°6′32.61″ | 94°40′3.68″ | 1138 |
| **6** |  | SSG | Shashangou, Kumtag | 39°39′51.46″ | 94°22′0.33″ | 1631 |
| **7** |  | AKS | Akesai, Kumtag | 39°25′14.52″ | 94°12′36.76″ | 2539 |
| **8** |  | JST | Jinshatan, Takalamakan | 42°2′55.32″ | 87°9′41.58″ | 1033 |
| **9** |  | DL | Dulan, Qaidam | 36°25′25.77″ | 98°7′25.35″ | 3123 |
| **10** |  | TGX | Tiegaixiang, Qaidam | 36°10′02.09″ | 100°34′13.85″ | 2905 |
| **11** |  | QHH | Qinghaihu, Qaidam | 36°43′3.13″ | 100°47′17.46″ | 3251 |
| **12** |  | M4 | M4, Badan Jaran | 40°38′12.46″ | 101°25′52.09″ | 1110 |
| **13** |  | S136 | S136, Badan Jaran | 39°43′49.14″ | 100°33′19.35″ | 1369 |
| **14** |  | M1 | M1, Badan Jaran | 39°38′8.81″ | 101°29′10.37″ | 1407 |
| **15** |  | AYQ | Ayouqi,Badan Jaran | 39°15′6.45″ | 101°40′52.02″ | 1523 |
| **16** |  | WLJ | Wuliji,Badan Jaran | 40°38′45.34″ | 104°33′36.49″ | 1253 |
| **17** |  | MQ | Minqin, Tengger | 38°53′35.02″ | 103°20′18.56″ | 1334 |
| **18** |  | TGL | Tengger , Tengger | 37°34′26.82″ | 105°1′15.97″ | 1276 |
| **19** |  | YLH | Yuelianghu,Tengger | 38°44′45.83″ | 105°21′19.76″ | 1286 |
| **20** |  | JLT | Jilantai, Ulan Buh | 39°55′40.01″ | 105°40′34.77″ | 1021 |
| **21** |  | BYWS | Bayinwusu, Ulan Buh | 39°56′31.40″ | 108°34′46.59″ | 1266 |
| **22** |  | SSLL | Sishililiang, Ulan Buh | 39°24'2.772" | 108°34'42.492" | 1537 |
| **23** |  | BLG | Balagong,Kubuqi | 40°15′42.68″ | 107°2′6.34″ | 1072 |
| **24** |  | YLJT | Yilijituan, Kubuqi | 41°45'6.779" | 107°0'22.921" | 1386 |
| **25** |  | HJNE | Hangjinnaoer,Kubuqi | 40°29′9.21″ | 108°51′32.04″ | 1045 |
| **26** |  | HJQ | Hangjinqi,Kubuqi | 39°51′0.97″ | 109°17′0.81″ | 1395 |
| **27** |  | DLZ | Daluzhen, Kubuqi | 40°2'19.788" | 111°10'11.568" | 1110 |
| **28** |  | WSQ | Wushenqi, Mu Us | 38°33′23.36″ | 108°51′6.05″ | 1279 |
| **29** |  | MMH | Miaomiaohu, Mu Us | 38°50′30.30″ | 106°49′34.02″ | 1270 |
| **30** |  | JB | Jingbian, Mu Us | 37°37′20.05″ | 108°44′16.33″ | 1336 |
| **31** |  | BB | Baobian, Mu Us | 37°36′50.84″ | 107°36′14.19″ | 1354 |
| **32** |  | SQL | Mu Us | 37°24'34.38" | 107°13'21.432" | 1358 |
| **33** |  | BYWZ | Bayinwuzhu, Mu Us | 38°24'1.944" | 107°26'45.6" | 1349 |
| **34** |  | EQQ | E’qianqi, Mu Us | 37°34'23.16" | 107°18'46.512" | 1343 |
| **35** | East | SGDL | Sanggendalai, Hunshandake | 43°7'20.748" | 116°5'58.128" | 1313 |
| **36** |  | HDJT | Hadajituan, Hunshandake | 42°31'44.22" | 115°36'43.679" | 1324 |
| **37** |  | KZYQ | Kezhongyouqi, Horqin | 45°1'42.842" | 121°34'7.979" | 236 |
| **38** |  | XBEH | Xinbaerhu, Hulun Buir | 48°58'28.02" | 119°58'1.308" | 653 |

**Table S4 Pairwise genetic differentiation (*F*_ST_) among 4 groups estimated from the neutral SNPs.**

|  | BEJ | Central_1 | Central_2 | East |
| --- | --- | --- | --- | --- |
| BEJ | 0.00 | - | - | - |
| Central_1 | 0.28 | 0.00 | - | - |
| Central_2 | 0.28 | 0.01 | 0.00 | - |
| East | 0.27 | 0.17 | 0.17 | 0.00 |

**Table S5 List of the bioclimatic variables used to develop the ecological niche models and the variable contributions for each group.**

| Lineage | Variable | Percent contribution | Permutation importance | Discription |
| --- | --- | --- | --- | --- |
| *Central* | bio_19 | 36.2 | 34.8 | Precipitation of Coldest Quarter |
|  | bio_11 | 23.2 | 34.5 | Mean Temperature of Coldest Quarter |
|  | bio_9 | 9.3 | 5.8 | Mean Temperature of Driest Quarter |
|  | bio_5 | 8.3 | 5.7 | Max Temperature of Warmest Month |
|  | bio_13 | 6.7 | 1.7 | Precipitation of Wettest Month |
|  | bio_18 | 6.2 | 7 | Precipitation of Warmest Quarter |
|  | bio_8 | 4.6 | 0.5 | Mean Temperature of Wettest Quarter |
|  | bio_4 | 2.6 | 0.7 | Temperature Seasonality  (standard deviation *100) |
|  | bio_2 | 2 | 6.1 | Mean Diurnal Range  (Mean of monthly (max- min temp)) |
|  | bio_15 | 0.9 | 2.9 | Precipitation Seasonality  (Coefficient of Variation) |
|  | bio_3 | 0 | 0.1 | Isothermality (BIO2/BIO7) (* 100) |
|  | bio_14 | 0 | 0 | Precipitation of Driest Month |
| *East* | bio_15 | 37.5 | 0 | Precipitation Seasonality  (Coefficient of Variation) |
|  | bio_9 | 22.5 | 6.9 | Mean Temperature of Driest Quarter |
|  | bio_3 | 17.2 | 20 | Isothermality (BIO2/BIO7) (* 100) |
|  | bio_14 | 8.9 | 56.4 | Precipitation of Driest Month |
|  | bio_18 | 4.3 | 12.5 | Precipitation of Warmest Quarter |
|  | bio_19 | 4.2 | 0 | Precipitation of Coldest Quarter |
|  | bio_11 | 2.9 | 4.1 | Mean Temperature of Coldest Quarter |
|  | bio_13 | 1.4 | 0 | Precipitation of Wettest Month |
|  | bio_2 | 1 | 0.1 | Mean Diurnal Range  (Mean of monthly (max- min temp)) |
|  | bio_4 | 0 | 0 | Temperature Seasonality  (standard deviation *100) |
|  | bio_5 | 0 | 0 | Max Temperature of Warmest Month |
|  | bio_8 | 0 | 0 | Mean Temperature of Wettest Quarter |


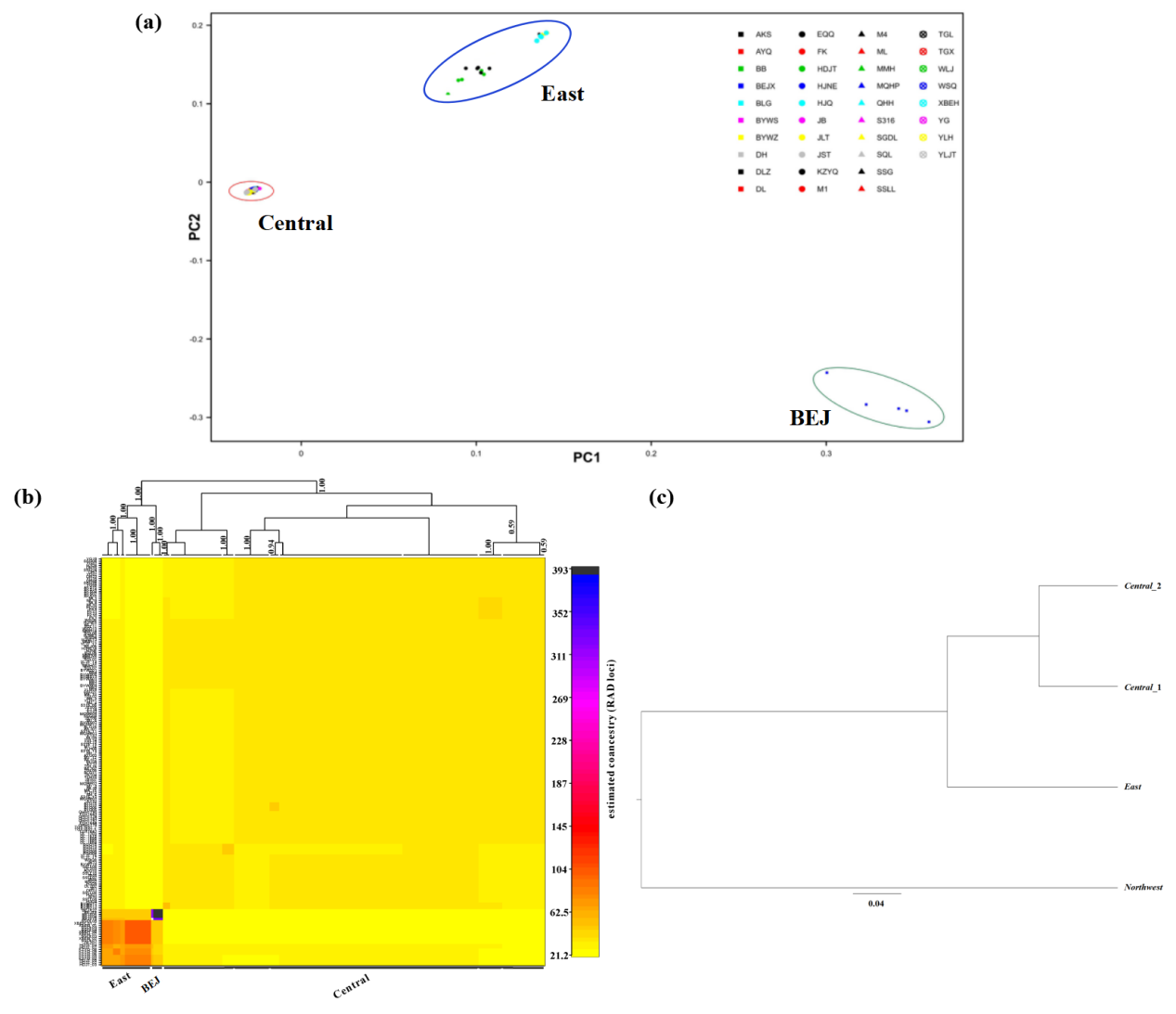


Fig. S1 Results of PCA, FineRADStrucutre and *Beast for sand rice populations.
